# Partial otubain 1 deficiency compromises fetal well-being in allogeneic pregnancies despite no major changes in the dendritic cell and T cell compartment

**DOI:** 10.1186/s13104-022-06230-w

**Published:** 2022-11-05

**Authors:** Annika Stutz, Gopala Nishanth, Ana C. Zenclussen, Anne Schumacher

**Affiliations:** 1 Experimental Obstetrics and Gynecology, Medical Faculty, Otto-von-Guericke University, Gerhart-Hauptmann-Straße 35, 39108 Magdeburg, Germany; 2 Institute of Medical Microbiology and Hospital Hygiene, Otto-von-Guericke University, Leipziger Straße 44, 39120 Magdeburg, Germany; 3 Institute of Medical Microbiology and Hospital Epidemiology, Hannover Medical School, Carl-Neuberg-Straße 1, 30625 Hannover, Germany; 4 Department of Environmental Immunology, Helmholtz Centre for Environmental Research - UFZ, Permoserstraße 15, 04318 Leipzig, Germany; 5 Perinatal Immunology, Saxonian Incubator for Clinical Translation (SIKT), Philipp-Rosenthal-Straße 55, 04103 Leipzig, Germany

**Keywords:** Deubiquitinating enzyme, Otubain 1, Dendritic cells, T cells, Fetal tolerance, Pregnancy

## Abstract

**Objective:**

Pregnancy is characterized by well-defined immunological adaptions within the maternal immune cell compartment allowing the survival of a genetically disparate individual in the maternal womb. Phenotype and function of immune cells are largely determined by intracellular processing of external stimuli. Ubiquitinating and deubiquitinating enzymes are known to critically regulate immune signaling either by modulating the stability or the interaction of the signaling molecules. Accordingly, if absent, critical physiological processes may be perturbed such as fetal tolerance induction. Based on previous findings that mice hemizygous for the deubiquitinating enzyme otubain 1 (OTUB1) do not give rise to homozygous progeny, here, we investigated whether partial OTUB1 deficiency influences fetal-wellbeing in a syngeneic or an allogeneic pregnancy context accompanied by changes in the dendritic cell (DC) and T cell compartment.

**Results:**

We observed increased fetal rejection rates in allogeneic pregnant OTUB1 heterozygous dams but not syngeneic pregnant OTUB1 heterozygous dams when compared to OTUB1 wildtype dams. Fetal demise in allogeneic pregnancies was not associated with major changes in maternal peripheral and local DC and T cell frequencies. Thus, our results suggest that OTUB1 confers fetal protection, however, this phenotype is independent of immune responses involving DC and T cells.

**Supplementary Information:**

The online version contains supplementary material available at 10.1186/s13104-022-06230-w.

## Introduction

Pregnancy is considered as a physiological state of immune tolerance towards the foreign paternal alloantigens expressed by fetal tissues. Establishment and maintenance of fetal tolerance depends on fine regulations of innate and adaptive immune processes. Consequently, immunological maladaptions result in a variety of pregnancy complications that can severely harm the mother and/or the fetus [1]. Particularly, Dendritic cells (DC) and T cells are major players in the immune regulatory network ensuring fetal well-being. Depending on their subtype, phenotype and function, these two immune cell populations determine fetal survival or rejection [2, 3]. Immune cell phenotypes and activities are commonly regulated by the availability and nature of certain cytokines, growth factors and hormones that are provided by the surrounding environment. However, immune cell fate is not restricted to extracellular signals but is also dictated by intracellular signaling molecules and by the pathways which they are part of. The stability and the interaction of signaling molecules with each other, in turn, is regulated by post-translational mechanisms including the ubiquitination/deubiquitination system. Ubiquitination is a process in which small regulatory proteins called ubiquitins are tagged onto lysine residues of a substrate protein. Depending upon the type of ubiquitin linkage, the substrate protein is either degraded by the proteasome or its interaction with other proteins is altered. Deubiquitinating enzymes (DUBs) operate as antagonists of the ubiquitination process. Among the DUBs, the ovarian tumour-related (OTU) proteases represent the second largest mammalian DUB family including Otubain 1 and 2 (OTUB1 and 2) [4]. OTUB1, the OTU domain-containing ubiquitin aldehyde-binding protein 1, was described to possess a pivotal role in cancer induction and progression [5]. With regard to pregnancy, Pasupala and colleagues reported that the homozygous deletion of OTUB1 induced lethality in late stages of embryonic development in mice [6]. Own previous observation showed that heterozygous OTUB1 interbreedings do not yield homozygous progeny [7]. With respect to immune cells, we and others showed that OTUB1 fostered DC activation and IL-12 production [8] and reduced T cell sensitization [9]. Whether this is also true during pregnancy has not been studied. In our study, we wondered whether partial OTUB1 deficiency results in fetal demise in a syngeneic or allogeneic pregnancy setting by perturbing peripheral or local DC and T cell populations in the maternal compartment.

## Methods

### Animals

Wildtype OTUB1^+/+^ and heterozygous OTUB1^+/−^ female and male mice (C57BL/6 background) were generated in Prof. Schlüter´s lab as previously described [7] and maintained by heterozygous interbreedings in an animal facility of the University Magdeburg, Germany. Non-mated mice were kept in groups in cages with enrichment under a 12 h light/12 h dark cycle at 22 ± 2 °C and an air humidity of 40–60%. Water and food were provided ad libitum. Homozygous OTUB1^−/−^ fetuses died intrauterine, thus preventing the inclusion of totally OTUB1-deficient mice in our analyses. Syngeneic mating combinations were set up by pairing 8-weeks old heterozygous OTUB1^+/−^ females to 8-weeks old heterozygous OTUB1^+/−^ males. Wildtype interbreedings, set up in parallel, served as controls. Age-matched allogeneic matings consisted of either heterozygous OTUB1^+/−^ or wildtype OTUB1^+/+^ females mated to wildtype BALB/c males that were purchased from Janvier Labs (France). After mating, dams were checked twice a day for a vaginal plug. Plug date was considered to be day 0 of gestation.

### Determination of pregnancy outcome, tissue sampling and processing

At gestation day (gd)12, blood was obtained by retroorbital puncture from all dams under anesthesia. Thereafter, dams were sacrificed by cervical dislocation, the abdomen was opened and the bicornial uterus was removed. Both uterine horns were opened longitudinally and the total number of viable and resorbed fetuses was recorded. Resorption sites were defined as necrotic and hemorrhagic tissue residues. Thymus, spleen and draining lymph nodes (inguinal and paraaortic) were collected and immediately transferred to ice-cold Roswell Park Memorial Institute (RPMI) 1640 medium (ThermoFisher, Germany). Uterine tissue was cut into pieces and digested for 90 min in RPMI 1640 medium supplemented with 5 % penicillilin/streptomycin (P/S; ThermoFisher, Germany) and 50 µg/ml liberase TL (Sigma, Germany) in an incubator at 37 °C and 5 ;% CO_2_. Digestion reaction was stopped by adding RPMI 1640 medium supplemented with 10 % fetal bovine serum (FBS, Biochrom, Germany) and 1 % P/S (referred as complete medium). Uterine tissue pieces were mashed through 100 μm and 40 μm cell strainers (Corning, USA) using complete medium to obtain single cell suspensions. Cell suspensions were further incubated for 30 min at 37 °C and 5 % CO_2_, were centrifuged and finally resuspended in complete medium. Thymus, spleen and lymph nodes were passed through a 100 μm cell strainer, incubated with erythrocyte lysis buffer (including blood samples), washed with complete medium, centrifuged and kept in complete medium for further analyses.

### Flow cytometry analyses

Extra- and intracellular antibody staining was performed to assess the frequencies of DC and T cell populations in lymphoid tissues, blood and uterine tissue. Briefly, cells were suspended in flow cytometry (FC) buffer containing PBS, 1 % bovine serum albumin (Merck Millipore, Germany) and 0.1 % sodium azide (Sigma, Germany). Afterwards, staining for extracellular markers (CD4, CD8, CD11c, MHCII, CD80) was performed for 30 min at 4 °C in the dark. Following a washing step in FC buffer, cells were fixed over night at 4 °C in the dark. Next day, cells were washed in permeabilization buffer and regulatory T cells (Treg) were defined by intracellular staining for the transcription factor FOXP3 for 30 min at 4 °C in the dark. For fixation and permeabilisation, the Fixation/Permeabilisation buffer set from ThermoFisher, Germany was used. Thereafter, the cells were washed in permeabilization buffer, resuspended in FC buffer, measured on a FACSCalibur (BD Biosciences, Germany) and analyzed using FlowJo V8 software (BD Biosciences, Germany). The following antibodies were applied: FITC-labeled anti-mouse CD4 (clone: RM4-4), PE-labeled anti-mouse FOXP3 (clone: NRRF-30); PE-Cy5.5-labeled anti-mouse CD8 (clone: 53 − 6.7), FITC-labeled anti-mouse CD80 (clone: 16-10A1), PE-labeled anti-mouse A-I/E-I (clone: M5/114.15.2) and APC-labeled anti-mouse CD11c (clone: HL3). All antibodies except for FOXP3 (eBioscience, Germany) were purchased from BD Biosciences, Germany. Exemplary FC dot plots of all analyzed DC and T cell populations in different tissues are displayed in Additional File 1.

### Data analysis and statistics

Data analysis was conducted with GraphPad Prism 7.0 software (Statcon, Germany). All data sets were analyzed for normal distribution using the Shapiro-Wilk test. Pregnancy outcome data for syngeneic and allogeneic pregnancies were analyzed using the chi square test. Flow cytometry data of DC and T cell populations were evaluated by using the non-parametric Mann-Whitney *U*-test. In all cases, p ≤ 0.05 was considered to be statistically significant.

## Results

### Partial OTUB1 deficiency induced fetal demise in an allogeneic but not in a syngeneic pregnancy setting

**Fig. 1:**
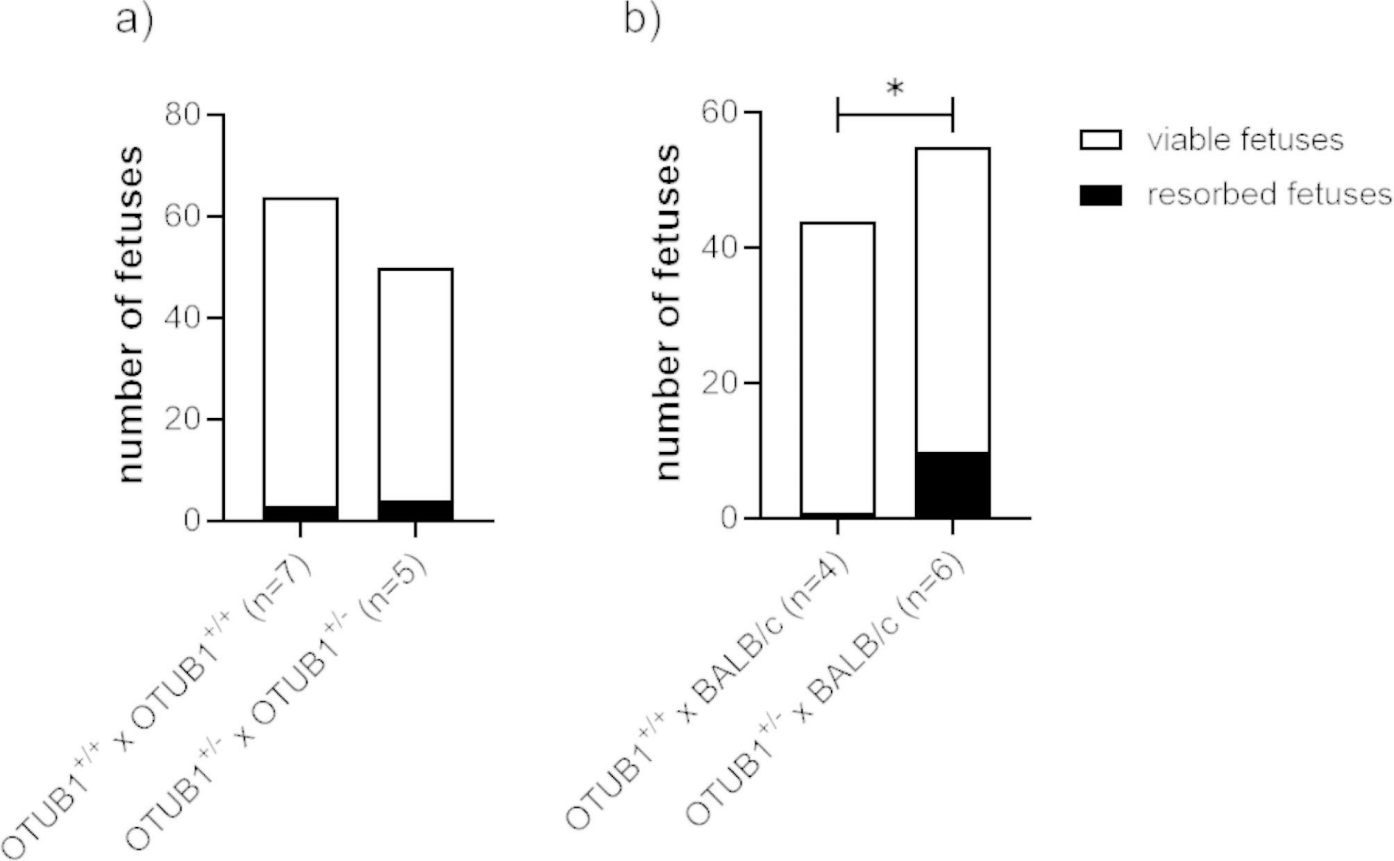
Partial OTUB1 deficiency provoked fetal rejection in allogeneic but not syngeneic pregnancies (a) Number of all fetuses (viable plus resorbed) from either syngeneic wildtype OTUB1^+/+^ interbreedings (n = 7) or syngeneic heterozygous OTUB1^+/−^ interbreedings (n = 5) are displayed. (b) Number of all fetuses (viable plus resorbed) from either allogeneic BALB/c-mated wildtype OTUB1^+/+^ dams (n = 4) or allogeneic BALB/c-mated heterozygous OTUB1^+/−^ dams (n = 6) are displayed. Data are presented as medians and statistical difference was assessed between the wildtype and heterozygous groups by chi square test. *p < 0.05

First, we studied the consequences of a lack of OTUB1 in syngeneic pregnancies by comparing the pregnancy outcome of heterozygous interbreedings with wildtype interbreedings. Partial OTUB1 deficiency in both parental sexes did not provoke significant differences in the total number of fetuses (implantations) and the number of resorbed fetuses (Fig. 1a). Second, we wondered whether a partial OTUB1 deficit in the dams would lead to more pronounced effects in a physiological pregnancy setting, meaning an allogeneic pregnancy. Indeed, we observed a significant increased number of resorbed fetuses in allogeneic pregnant heterozygous OTUB1^+/−^ dams as compared to allogeneic pregnant wildtype OTUB1^+/+^ dams with total number of fetuses being not affected (Fig. 1b).

### Partial OTUB1 deficiency altered CD4^+^ T cell frequencies but not DC frequencies in syngeneic pregnancies

Determinations of alterations of peripheral and local DC frequencies in response to a partial OTUB1 deficit in both parental sexes revealed no changes in total DC frequencies or frequencies of mature DC populations within all organs studied (Table 1). By contrast, frequencies of total CD4^+^ T cells were significantly elevated in blood and uterine tissue from heterozygous interbreedings when compared to wildtype interbreedings (Table 1). Notably, CD4^+^FOXP3^+^ Treg frequencies were significantly decreased in thymus and significantly augmented in uterine tissue obtained from heterozygous dams (Table 1). No significant changes were observed in total CD8^+^ T cell frequencies between both mating combinations (Table 1).

**Table 1:** Partial OTUB1 deficiency altered T cell but not DC frequencies in syngeneic pregnancies

DC populations	thymus	spleen	lymph nodes	blood	uterus
**total CD11c** ^**+**^					
OTUB1^+/+^ x OTUB1^+/+^	0.13 ± 1.18	2.49 ± 2.86	0.60 ± 0.61	3.12 ± 2.29	1.19 ± 0.93
OTUB1^+/−^ x OTUB1^+/−^	0.39 ± 0.20	4.19 ± 0.89	0.82 ± 0.31	2.19 ± 1.06	0.85 ± 0.73
**mature CD11c** ^**+**^ **CD80** ^**+**^					
OTUB1^+/+^ x OTUB1^+/+^	0.01 ± 0.09	0.32 ± 0.60	0.07 ± 0.04	0.63 ± 0.79	0.25 ± 0.14
OTUB1^+/−^ x OTUB1^+/−^	0.04 ± 0.05	1.03 ± 0.41	0.06 ± 0.03	0.85 ± 0.37	0.15 ± 0.09
**mature CD11c** ^**+**^ **MHCII** ^**+**^					
OTUB1^+/+^ x OTUB1^+/+^	0.05 ± 0.31	1.24 ± 1.13	0.22 ± 0.26	0.28 ± 0.31	0.54 ± 0.47
OTUB1^+/−^ x OTUB1^+/−^	0.11 ± 0.08	1.22 ± 0.16	0.36 ± 0.11	0.18 ± 0.21	0.28 ± 0.51
**total CD8** ^**+**^					
OTUB1^+/+^ x OTUB1^+/+^	85.88 ± 11.83	12.63 ± 3.87	22.65 ± 4.30	18.18 ± 7.14	1.08 ± 0.37
OTUB1^+/−^ x OTUB1^+/−^	92.34 ± 2.73	16.63 ± 2.66	25.19 ± 4.10	24.80 ± 2.98	0.37 ± 0.51
**total CD4** ^**+**^					
OTUB1^+/+^ x OTUB1^+/+^	88.30 ± 9.53	12.38 ± 3.17	25.31 ± 4.89	20.48 ± 9.29	0.77 ± 1.26
OTUB1^+/−^ x OTUB1^+/−^	95.74 ± 1.74	14.10 ± 6.52	29.34 ± 4.94	29.88 ± 8.43*	4.93 ± 1.98*
**CD4** ^**+**^ **Foxp3** ^**+**^ **Treg**					
OTUB1^+/+^ x OTUB1^+/+^	1.34 ± 0.94	1.77 ± 0.76	3.64 ± 1.11	1.55 ± 1.01	0.06 ± 0.05
OTUB1^+/−^ x OTUB1^+/−^	0.52 ± 0.27*	1.86 ± 0.40	3.69 ± 0.85	2.27 ± 0.55	0.13 ± 0.12*

Peripheral and local DC and T cell populations were analyzed in heterozygous (n = 5) and wildtype (n = 7) interbreedings. Data is presented as medians plus standard deviation. Statistical difference between both mating combinations was assessed by using the non-parametric Mann-Whitney *U*-test. *p < 0.05.

### Partial OTUB1 deficit affected total splenic CD4^+^ T cell frequencies but did not change DC frequencies in allogeneic pregnancies

In allogeneic pregnancies, maternal immune cells are particularly challenged by the presence of foreign fetal antigens and inadequate maternal immune responses often cause fetal demise. As we observed increased fetal resorptions in allogeneic pregnant heterozygous OTUB1^+/−^ dams, we speculated that partial OTUB1 deficiency lead to disturbances in the peripheral and local maternal DC and T cell compartments, thereby inducing fetal rejection. However, our analyses revealed no significant changes in peripheral and local DC and T cell frequencies with the exception of a significant increase in total splenic CD4^+^ T cells of allogeneic pregnant heterozygous OTUB1^+/−^ dams as compared to allogeneic pregnant wildtype OTUB1^+/+^ dams (Table 2).

**Table 2:** Partial OTUB1 deficiency altered splenic CD4^+^ T cell but not DC frequencies in allogeneic pregnancies

DC populations	thymus	spleen	lymph nodes	blood	uterus
**total CD11c** ^**+**^					
OTUB1^+/+^ x BALB/c	0.81 ± 0.57	2.18 ± 0.55	1.36 ± 0.47	2.23 ± 0.48	2.82 ± 1.03
OTUB1^+/−^ x BALB/c	0.83 ± 0.51	2.42 ± 1.07	1.90 ± 0.64	2.61 ± 1.81	2.27 ± 1.34
**mature CD11c** ^**+**^ **CD80** ^**+**^					
OTUB1^+/+^ x BALB/c	0.01 ± 0.03	0.33 ± 0.21	0.07 ± 0.02	0.47 ± 0.85	0.34 ± 0.12
OTUB1^+/−^ x BALB/c	0.02 ± 0.04	0.27 ± 0.24	0.03 ± 0.02	0.41 ± 0.46	0.24 ± 0.24
**mature CD11c** ^**+**^ **MHCII** ^**+**^					
OTUB1^+/+^ x BALB/c	0.12 ± 0.07	0.83 ± 0.23	0.56 ± 0.11	0.25 ± 0.12	1.43 ± 0.59
OTUB1^+/−^ x BALB/c	0.12 ± 0.10	0.95 ± 0.56	0.36 ± 0.14	0.29 ± 0.19	1.27 ± 0.33
**total CD8** ^**+**^					
OTUB1^+/+^ x BALB/c	92.90 ± 3.89	9.05 ± 3.90	21.45 ± 7.85	21.05 ± 3.49	1.95 ± 1.97
OTUB1^+/−^ x BALB/c	90.40 ± 5.45	12.15 ± 2.34	25.30 ± 4.53	22.95 ± 3.31	0.81 ± 1.99
**total CD4** ^**+**^					
OTUB1^+/+^ x BALB/c	97.17 ± 2.17	11.42 ± 1.78	21.24 ± 5.10	22.68 ± 7.77	5.53 ± 1.70
OTUB1^+/−^ x BALB/c	95.91 ± 4.84	14.44 ± 1.38*	25.68 ± 3.96	25.93 ± 8.77	3.44 ± 2.23
**CD4** ^**+**^ **Foxp3** ^**+**^ **Treg**					
OTUB1^+/+^ x BALB/c	0.87 ± 0.63	1.78 ± 0.63	3.38 ± 0.56	1.42 ± 0.65	0.14 ± 0.12
OTUB1^+/−^ x BALB/c	0.81 ± 0.65	1.63 ± 0.68	2.87 ± 0.55	1.71 ± 0.91	0.26 ± 0.21

Peripheral and local DC and T cell populations were analyzed in BALB/c-mated heterozygous OTUB1^+/−^ dams (n = 6) and BALB/c-mated wildtype OTUB1^+/+^ dams (n = 4). Data is presented as medians plus standard deviation. Statistical difference between both mating combinations was assessed by using the non-parametric Mann-Whitney *U*-test. *p < 0.05

## Discussion

Ubiquitination and deubiquitination of proteins are two opposed processes allowing a timely and well-coordinated regulation of the intracellular signaling cascade. As ubiquitinating and deubiquitinating enzymes target a variety of substrates, they critically intervene with a plethora of physiological mechanisms. In this sense, the absence of one enzyme can have already dramatic consequences for critical life-sustaining processes such as fetal survival in the maternal womb. During the generation of a transgenic mouse line fully devoid of the DUB OTUB1, Prof. Schlüter´s lab became aware that heterozygous OTUB1 interbreedings do yield homozygous progeny. This is in agreement with observations made by Pasupala and colleagues who found 35 % wildtype OTUB1 progeny, 65 % heterozygous OTUB1 progeny and 0 % homozygous OTUB1 progeny out of 124 live born mice of heterozygous interbreedings. Analyses at gd14.5 revealed that the mutant OTUB1^−/−^ allele segregated in perfect Mendelian ratio whereupon the authors concluded that complete OTUB1 deficiency causes lethality at late embryonic stages. They further assumed that OTUB1 deficiency might cause embryonic lethality by impairing ubiquitination of its target molecules and thereby interfere with mechanisms essential for late embryogenesis [6]. We showed that syngeneic heterozygous interbreedings did not suffer from elevated fetal rejection rates at gd12 and individual fetuses showed no macroscopic differences among each other. Unfortunately, we were not successful in obtaining sufficient non-degraded fetal DNA to perform genotypic analyses of each individual fetus at gd12. In agreement with our findings, Pasupala and colleagues did not mention any abnormalities of their homozygous OTUB1 embryos at gd14.5 [6] and suggested rejection of homozygous OTUB1 fetuses taking place at later pregnancy stages. Moreover, we wondered whether a lack of OTUB1 on the maternal side would have any consequences for fetal well-being in a more physiological pregnancy setting, namely an allogeneic pregnancy. As OTUB1 was described to modulate DC and T cell functions [10–13] we hypothesized that its loss may provoke perturbances in maternal immune responses and thereby interfere with fetal tolerance induction. Indeed, we observed a significant increased number of resorbed fetuses in allogeneic pregnancies where dams showed a partial OTUB1 deficiency. However, fetal demise does not seem to be driven by major immunological changes in the periphery and locally at the fetal-maternal interface, at least not in the DC and T cell compartment. This assumption is further underlined by our observation that dysregulated total CD4^+^ T cell and Treg frequencies in the periphery and in the uterine tissue in syngeneic OTUB1 heterozygous interbreedings were not associated with increased fetal rejection rates. Treg play an essential role in fetal tolerance induction, particularly in allogenic pregnancies. Since the transcription factor NF-κB is essential for the stability and activation of Treg [14] and our previous study showed that OTUB1 augments NF-κB-dependent responses in DC [8], one could hypothesize that the reduced activation of NF-κB in Treg of OTUB1^+/−^ mice might lead to impaired Treg function and thereby result in increased numbers of resorbed fetuses in allogeneic pregnant OTUB1^+/−^ dams. Moreover, it remains to be elucidated in future studies whether other immune cells than DC and T cells are negatively affected by a lack of OTUB1 and may explain fetal demise in allogeneic pregnancies. For instance, OTUB1 has been shown to control the maturation and activation of Natural Killer cells [9], known to regulate critical steps during fetal development [15, 16].

In conclusion, our results provide evidence that OTUB1 is involved in fetal protection in allogeneic pregnancies. This protective effect seems not to be mediated through modulation of maternal DC and T cell responses. However, whether OTUB1 influences other immune cell populations or other factors essential for fetal survival merits further investigation.

## Limitations

Our current study is limited by some aspects in the study design and technical issues such as a partial OTUB1 deficit in both parental sexes in an allogeneic pregnancy setting, the inclusion of further innate and adaptive immune cell populations in the analyses and lack of information on fetal genotype due to inappropriate sample preservation. However, the setup of an allogeneic pregnancy model yielding in homozygous OTUB1 fetuses is very time consuming as well as detailed immunological analyses and goes beyond the scope of the present study.

## Electronic supplementary material

Below is the link to the electronic supplementary material.

Supplementary Material 1. Representative flow cytometry dot plots are displayed for different DC and T cell populations within each organ analyzed. First, total lymphocytes were gated. Then, DC and T cell frequencies were determined according to their specific marker combinations

## Data Availability

The datasets supporting the conclusions of this article are included within the article and its additional file.

## List of abbreviations

DC: dendritic cell
DUB: deubiquitylating enzyme
FBS: fetal bovine serum
FC: flow cytometry
FOXP3: forkhead box protein P3:gd, gestation day
P/S: penicillin/streptomycin
RPMI: Roswell Park Memorial Institute
OTUB1: ovarian tumor-related domain-containing ubiquitin aldehyde-binding protein 1
Treg: regulatory T cell

## References

[1] Orefice R. Immunology and the immunological response in pregnancy. Best practice & research Clinical obstetrics & gynaecology. 2021;76:3–12. 10.1016/j.bpobgyn.2020.07.013 33191116

[2] Wei R, Lai N, Zhao L, Zhang Z, Zhu X, Guo Q, et al. Dendritic cells in pregnancy and pregnancy-associated diseases. 133: Biomedicine & pharmacotherapy = Biomedecine & pharmacotherapie; 2021. p. 110921. 10.1016/j.biopha.2020.110921 33378991

[3] Eikmans M, van der Zwan A, Claas FHJ, van der Hoorn ML, Heidt S (2020). Got your mother in a whirl: The role of maternal T cells and myeloid cells in pregnancy. Hla.

[4] Nijman SM, Luna-Vargas MP, Velds A, Brummelkamp TR, Dirac AM, Sixma TK (2005). A genomic and functional inventory of deubiquitinating enzymes. Cell.

[5] Saldana M, VanderVorst K, Berg AL, Lee H, Carraway KL (2019). Otubain 1: a non-canonical deubiquitinase with an emerging role in cancer. Endocrine-related Cancer.

[6] Pasupala N, Morrow ME, Que LT, Malynn BA, Ma A, Wolberger C (2018). OTUB1 non-catalytically stabilizes the E2 ubiquitin-conjugating enzyme UBE2E1 by preventing its autoubiquitination. J Biol Chem.

[7] Dong W, Wang H, Shahzad K, Bock F, Al-Dabet MM, Ranjan S (2015). Activated Protein C Ameliorates Renal Ischemia-Reperfusion Injury by Restricting Y-Box Binding Protein-1 Ubiquitination. J Am Soc Nephrology: JASN.

[8] Mulas F, Wang X, Song S, Nishanth G, Yi W, Brunn A (2021). The deubiquitinase OTUB1 augments NF-κB-dependent immune responses in dendritic cells in infection and inflammation by stabilizing UBC13. Cell Mol Immunol.

[9] Zhou X, Yu J, Cheng X, Zhao B, Manyam GC, Zhang L (2019). The deubiquitinase Otub1 controls the activation of CD8(+) T cells and NK cells by regulating IL-15-mediated priming. Nat Immunol.

[10] Xuan NT, Trung DM, Minh NN, Nghia VX, Giang NV, Canh NX (2019). Regulation of p38MAPK-mediated dendritic cell functions by the deubiquitylase otubain 1. Hla.

[11] Soares L, Seroogy C, Skrenta H, Anandasabapathy N, Lovelace P, Chung CD (2004). Two isoforms of otubain 1 regulate T cell anergy via GRAIL. Nat Immunol.

[12] Stempin CC, Rojas Marquez JD, Ana Y, Cerban FM (2017). GRAIL and Otubain-1 are Related to T Cell Hyporesponsiveness during Trypanosoma cruzi Infection. PLoS Negl Trop Dis.

[13] Lin JT, Lineberry NB, Kattah MG, Su LL, Utz PJ, Fathman CG, et al. Naive CD4 t cell proliferation is controlled by mammalian target of rapamycin regulation of GRAIL expression. Journal of immunology (Baltimore, Md: 1950). 2009;182(10):5919-28. 10.4049/jimmunol.0803986 PMC2853371 19414743

[14] Ronin E, Lubrano di Ricco M, Vallion R, Divoux J, Kwon HK, Grégoire S (2019). The NF-κB RelA Transcription Factor Is Critical for Regulatory T Cell Activation and Stability. Front Immunol.

[15] Meyer N, Woidacki K, Maurer M, Zenclussen AC (2017). Safeguarding of Fetal Growth by Mast Cells and Natural Killer Cells: Deficiency of One Is Counterbalanced by the Other. Front Immunol.

[16] Mahajan D, Sharma NR, Kancharla S, Kolli P, Tripathy A, Sharma AK, et al. Role of Natural Killer Cells during Pregnancy and Related Complications. Biomolecules. 2022;12(1). 10.3390/biom12010068 PMC8773865 35053216

