# Supplementary figures and images for: Partial otubain 1 deficiency compromises fetal well-being in allogeneic pregnancies despite no major changes in the dendritic cell and T cell compartment
Source: BMC Res Notes. 2022 Nov 5;15:341. doi: 10.1186/s13104-022-06230-w (PMC9636684; doi:10.1186/s13104-022-06230-w)

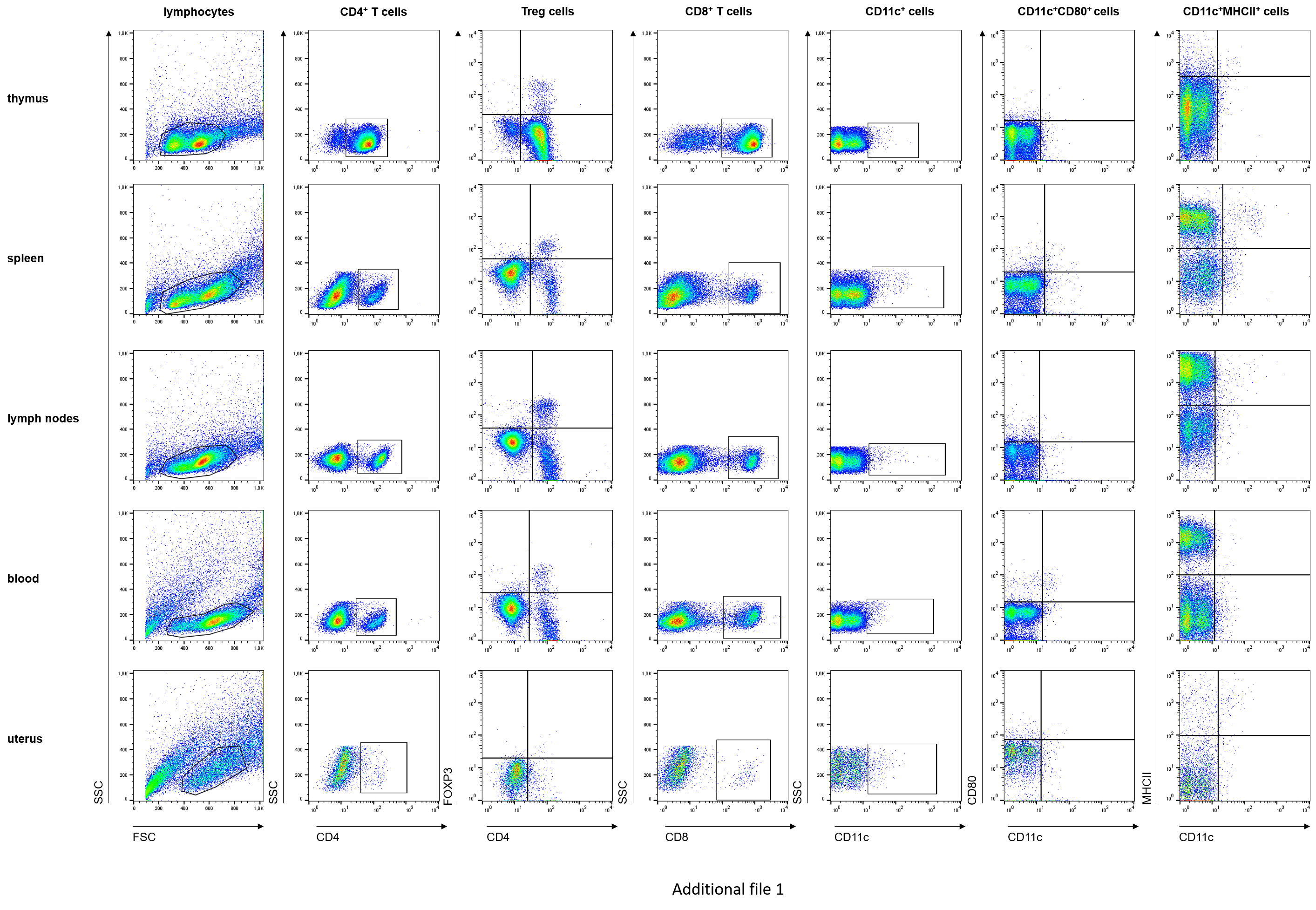

Supplement: Supplementary file 1 — Supplementary Material 1. Representative flow cytometry dot plots are displayed for different DC and T cell populations within each organ analyzed. First, total lymphocytes were gated. Then, DC and T cell frequencies were determined according to their specific marker combinations [file 13104_2022_6230_MOESM1_ESM.png]
